# COVID-19 Vaccine Hesitancy among Parents of Children and Adolescents Living in Brazil

**DOI:** 10.3390/vaccines9101115

**Published:** 2021-09-30

**Authors:** Leonardo Evangelista Bagateli, Edna Yayoi Saeki, Marta Fadda, Carlo Agostoni, Paola Marchisio, Gregorio Paolo Milani

**Affiliations:** 1 Department of Clinical Science and Community Health, Universita’ Degli Studi di Milano, 20122 Milan, Italy; leonardo.evangelistabagateli@studenti.unimi.it (L.E.B.); paola.marchisio@unimi.it (P.M.); milani.gregoriop@gmail.com (G.P.M.); 2 Pediatrics, Hospital Estadual de Bauru, Bauru 17033-360, Brazil; eysaeki@yahoo.com.br; 3 Facoltà di Scienze Biomediche, Università della Svizzera Italiana, 6900 Lugano, Switzerland; marta.fadda@usi.ch; 4 Pediatric Unit, Fondazione IRCCS Ca’ Granda Ospedale Maggiore Polcilinico, 20122 Milan, Italy

**Keywords:** SARS-CoV-2, low-income country, vaccination, children, adolescents, variants, barriers, Parent Attitudes about Childhood Vaccines

## Abstract

Background: The immunization of large portions of populations in low/middle-income countries is considered one of the key measures to limit the development of new SARS-CoV-2 variants. However, parental vaccine hesitancy might be an important obstacle to pediatric vaccination. The aim of this survey was to study the prevalence and extent of COVID-19 vaccine hesitancy among parents of children and adolescents living in Brazil. Methods: Caregivers of children and adolescents referred to the emergency department of Hospital Estadual de Bauru, São Paulo (Brazil) were invited to fill in a validated questionnaire on vaccine hesitancy and to report their willingness for themselves and their offspring to receive a COVID-19 vaccine. Results: A total of 501 consecutive caregivers filled in the survey. Response rate was 100%. A minority (N = 14, 2.8%) of caregivers were hesitant about vaccines. Despite this, half of them declared they were willing to vaccinate their offspring against COVID-19. Conclusions: This survey identifies that vaccine hesitancy is very low among caregivers living in Brazil and that even many of the hesitant caregivers are willing to vaccinate their offspring against COVID-19. This study highlights the importance of offering the COVID-19 vaccination to the whole population, including subjects that present uncertainty about other vaccines.

## 1. Introduction

Universal COVID-19 vaccination is considered the key measure to limit the spread of SARS-CoV-2 and the risk of the emergence of new variants [1,2]. The success of vaccination programs relies, among others, on the immunization of large portions of pediatric and adult populations in low/middle-income countries where SARS-CoV-2 variants of concern have been detected [3,4].

By 24 September 2021, a total of 85.3 million (~40%) people in Brazil had been fully vaccinated against COVID-19 [5]. Although vaccination coverage in this country—the largest of South and Latin America—is higher than the vaccination coverage of low-income countries (on average < 2.5%) [5], a significant amount of Brazilian children and adolescents have not yet received COVID-19 vaccination.

Parental vaccine hesitancy, defined as parents’ delay in acceptance or refusal of vaccines despite their availability for their children, might constitute an important obstacle to vaccination [6]. Yet, few studies investigated parental beliefs regarding COVID-19 vaccination and contrasting data are available. A survey conducted before the first COVID-19 vaccine authorizations in Western countries (December 2020) found that <50% of pregnant women were willing to vaccinate their child against COVID-19 in the USA and Australia. The same survey found that >50% of pregnant women were willing to vaccinate their children in countries with a lower income such as Brazil, Colombia and Mexico [7]. Different results were obtained in a survey conducted in Italy between December 2020 and January 2021: this study found that only a minority of parents (~10%) were hesitant toward a possible COVID-19 vaccination for their children [8]. On the contrary, surveys administered a few months after the implementation of COVID-19 vaccine programs found that about half of US parents were hesitant about COVID-19 vaccinations for their children [9,10]. Therefore, there is a need to provide updated results on vaccine beliefs and COVID-19 vaccine attitudes among parents worldwide.

The aim of this study was to investigate the prevalence and extent of COVID-19 vaccine hesitancy among parents of children and adolescents living in Brazil.

## 2. Materials and Methods

### 2.1. Study Design and Participant Recruitment

We invited all caregivers of children and adolescents referred to the emergency department of Hospital Estadual de Bauru, São Paulo (Brazil), to take part in a cross-sectional study between May and June, 2021. To be eligible, caregivers had to be able to speak Portuguese or English. Caregivers aged < 18 years were not included. A validated, structured questionnaire (“Parent Attitudes about Childhood Vaccines”) was employed [11]. Before administration, a pilot study was conducted to translate and adapt the questionnaire to Portuguese following standard procedures previously reported for other countries [12]. Briefly, the questionnaire was translated into Portuguese by one of the researchers (LEB). Then, it was administered twice with a one-week interval to a convenience sample of 2 pediatricians and 8 parents.

### 2.2. Survey Instrument

The “Parent Attitudes about Childhood Vaccines” questionnaire comprises 15 items related to childhood vaccination behavior (2 questions), safety and efficacy (4 questions) and general attitudes and trust (9 questions) about pediatric vaccinations. A numeric score ranging from 0 (not hesitant) to 2 (hesitant) was assigned for each of the 15 answers, according to previously reported scoring systems [12,13]. To quantify a vaccine hesitancy score, the score of each item was summed and converted to a 0–100 scale by means of a simple linear transformation. Participants with a score ≥ 50 were defined as “hesitant”. At the end of the questionnaire, participants were asked to answer (“yes”, “no” or “I don’t know”) two further questions about their willingness to have their child/adolescent vaccinated against COVID-19 (“Would you have your child vaccinated with a vaccine reported effective against COVID-19 and approved by the authorities?”) and themselves (“Would you have yourself vaccinated with a vaccine reported effective against COVID-19 and approved by the authorities?”). Finally, information on sex, age and ethnicity of the participants and their child, number of other siblings in the household, marital status, education level and household income was collected. The highest, completed formal education was considered to determine the education level.

### 2.3. Data Collection

To fill in the questionnaire, participants were invited to scan a QR code through their smartphone and answer an anonymous online questionnaire. In cases of children with one or more siblings, questions about vaccination attitudes, beliefs and willingness as well as all child-specific questions were focused on the child attending the emergency department. If more than one caregiver was present (e.g., both the mother and the father), only one of them could participate in the study. The time required to fill in the questionnaire was <15 min.

### 2.4. Data Analysis and Statistics

All data were prospectively collected. Categorical and ordinal data are presented as absolute and relative frequency, respectively. Fisher’s exact test or Chi-squared test were employed to compare data. Analyses were performed using the open-source statistical software R, Vienna, version 3.5.3 (11 March 2019).

### 2.5. Ethics

The study was approved by the institutional ethics committee. Written informed consent was provided by all participants.

## 3. Results

A convenience sample of 502 consecutive caregivers was invited to participate. Among the 502 caregivers, 1 subject aged <18 years was excluded. None of the remaining 501 subjects declined to participate (100% response rate). There were no missing data. The characteristics of the respondents are provided in Table 1.

The sample includes 426 (85%) mothers and 75 (15%) fathers. Most participants were 30 years old or older. The age of the child presenting to the emergency department was 8 or younger in more than half of the cases.

Supplementary Table S1 reports the results regarding beliefs and attitudes towards childhood vaccination. Although one in every five caregivers reported to have delayed a child vaccination for reasons other than illness or allergy, more than 90% of caregivers were confident that the recommended vaccination schedule was suitable for their child and almost all caregivers stated that if they had another infant today, they would have him/her vaccinated. On the other hand, 268 (53%) of the caregivers were concerned about serious side effects of the vaccines and 170 (34%) had some concerns about their safety. Overall, only 14 out 501 (2.8%) had a vaccine hesitancy score ≥ 50.

A total of 458 (91%) reported to be willing to accept COVID-19 vaccination for their child and a similar number of caregivers (N = 469, 94%) would get vaccinated against COVID-19. Twenty-three (4.6%) and 14 (2.8%) caregivers were undecided about COVID-19 vaccination for their child or for themselves, respectively. The remaining caregivers would not accept COVID-19 vaccination for their child (N = 20, 4.0%) and for themselves (N = 18, 3.6%). Interestingly, 7 out of the 14 (50%) hesitant caregivers would accept a vaccination against COVID-19 both for themselves and for their child. Four hesitant caregivers did not know if they would accept it, and three would not accept it for their child and themselves.

Caregivers’ young age (*p* < 0.001), the presence of two or more number of children in the house (*p* = 0.02), a lower educational level of the caregivers and having a low household income were associated with refusal of the COVID-19 vaccine for their children (Table 2).

## 4. Discussion

Vaccination against COVID-19 is currently the most effective intervention to reduce the burden of the COVID-19 pandemic. Although some degree of vaccine hesitancy was observed in many of the respondents, this survey points out that a large majority of caregivers living in Brazil were not hesitant about vaccinations. Moreover, the large majority would vaccinate their child against COVID-19.

Beliefs about vaccines and trust in health authorities are crucial during vaccination campaigns [14] and vaccine hesitancy has been widely documented in adult subjects [15,16]. This study shows for the first time that both vaccine hesitancy and refusal of vaccines against COVID-19 are very low among caregivers living in Brazil. It has been estimated that between 10% and 30% of parents are hesitant about vaccines in Western countries [8,17,18]. The findings of the present study confirm that middle-income countries have a lower tendency to vaccine hesitancy as compared to high-income countries [19]. On the other hand, it should be considered that the educational level of the caregivers in this survey was, on average, higher as compared with previous studies conducted in countries with limited resources [20].

No difference between male and female caregivers was found in relation to acceptance of COVID-19 vaccination. On the other hand, similar to other studies on vaccine hesitancy, we found an association between vaccine refusal and caregivers’ young age, low education level and household income [21,22,23]. These observations might contribute to the emerging picture of COVID-19 vaccine hesitancy and to develop tailored interventions to promote vaccine uptake [24].

An important finding of this survey is that even among hesitant caregivers, half of them stated they approved a vaccine against COVID-19 both for their child and themselves. We do not have a clear-cut explanation about this result. Yet, we speculate that three factors might underlie this observation: first, the overwhelming effect of the COVID-19 pandemic on Brazilian children might have played a role in the vaccine willingness of the caregivers [25]. Second, some parents might have a higher perception of risks associated with COVID-19 for their children as compared with other infections [26]. Finally, adults might be concerned that children and adolescents act as chain of transmission of SARS-CoV-2 infection in their families. Future studies should confirm and better explore our unexpected observation. In the meantime, the findings of this study have significant potential implications for Brazilian policymakers and healthcare providers. Since vaccination against COVID-19 might be accepted by the vast majority of parents, including those hesitant toward other vaccines, increasing efforts should be addressed to make the COVID-19 vaccination available to the whole population.

Most children affected by SARS-CoV-2 develop an asymptomatic or mild disease [27,28]. On the other hand, a minority of cases present more severe manifestations such as lower tract respiratory infections (e.g., bronchiolitis or pneumonia) or cardiac diseases, as in the case of multisystem inflammatory system [29,30,31]. Recent data showed that the risk of hospitalization for COVID-19 illness in children, including admission to intensive care units, is similar to that of influenza [32]. This risk is especially high in children with pre-existing chronic diseases, who are overall more vulnerable to severe complications of viral infections [33,34,35]. On the other hand, the burden of SARS-CoV-2 spread in the pediatric population should also be considered in view of further potential consequences that are not directly associated with the infection. Several studies showed that child and adolescent growth, development and mental health were negatively affected by the current pandemic [36,37,38,39]. Moreover, many data pointed out that school closures, which were adopted by many countries to contrast the pandemic [40], might lead to concerning effects on children’s wellbeing [41,42]. Finally, the “indirect” consequences of the COVID-19 pandemic among children and adolescents significantly impacted family wellbeing and the economy, and contributed to parental stress [43]. All these aspects together with the willingness of parents to vaccine their children further support the importance of ensuring COVID-19 vaccination access for children and adolescents worldwide [44,45].

This study has limitations. First, it is a single center study. Future studies should investigate the vaccine willingness of caregivers in other centers of Brazil and other middle-income countries. Second, we did not investigate if caregivers, their children or relatives had been previously infected by SARS-CoV-2. Third, similarly to previous studies on vaccine hesitancy [46,47], the survey was conducted in the waiting room of an emergency department. Therefore, we cannot rule out that the anxiety for the current status of the child might have transiently influenced the answers of the caregivers. However, previous investigations have shown that administration of the “Parent Attitudes about Childhood Vaccines” in emergency rooms is feasible and provides reliable data [48]. Two main strengths of this study should also be acknowledged. First, a validated questionnaire was used to evaluate vaccine hesitancy. Second, the response rate was extremely high.

## 5. Conclusions

This study shows that vaccine hesitancy is low among parents living in Brazil. Increasing efforts by policymakers and healthcare providers are needed to make vaccination available to the whole population, including parents hesitant toward other vaccinations. Future studies might provide new insights on the reasons for COVID-19 vaccine acceptance in subjects hesitant toward other vaccinations.

## Figures and Tables

**Table 1:** Characteristics of the caregivers (N = 501).

Characteristics	N (%)	
**Relationship to Child**		
Mother	426	(85)
Father	75	(15)
**Age (years)**		
18–29	67	(13)
30 or older	434	(87)
**Number of Children in Household**		
One	176	(35)
Two	231	(46)
Three or more	94	(19)
**Marital Status**		
Single, Widowed, Separated or Divorced	64	(13)
Married or Living with a partner	437	(87)
**Education**		
Up to High School incomplete	52	(10)
High School and/or Technical School complete	124	(25)
University incomplete or complete	127	(25)
Postgraduate incomplete or complete	198	(40)
**Household Income**		
Up to 3 minimum wage	190	(38)
From 3 to 6 minimum wages	103	(21)
6 or more minimum wages	208	(42)
**Race/Ethnicity**		
White	325	(65)
Non-white	176	(35)
**Child’s Age (years)**		
0–4	163	(33)
5–8	132	(26)
9–12	101	(20)
13–17	105	(21)
**Child’s Gender**		
Male	286	(57)
Female	215	(43)

**Table 2:** Factors associated with COVID-19 vaccine approval (N = 458) and refusal (N = 20) for children among caregivers.

	Yes		No		
Characteristics	N (%)		N (%)		*p* Value
**Relationship to Child**					
Mother	388	(81)	17	(3.6)	0.999
Father	70	(15)	3	(0.6)	
**Age (years)**					
18–29	51	(11)	10	(2.1)	<0.001
30 or older	407	(85)	10	(2.1)	
**Number of Children in Household**					
One	163	(34)	4	(0.8)	0.0179
Two	213	(45)	7	(1.5)	
Three or more	82	(17)	9	(1.9)	
**Marital Status**					
Single, Widowed, Separated or Divorced	58	(12)	5	(1)	0.2081
Married or Living with a partner	400	(84)	15	(3.1)	
**Education**					
Up to High School incomplete	42	(8.8)	7	(1.5)	<0.001
High School and/or Technical School complete	105	(22)	8	(1.7)	
University incomplete or complete	123	(26)	4	(0.8)	
Postgraduate incomplete or complete	188	(39)	1	(0.2)	
**Household Income**					
Up to 3 minimum wage	163	(34)	13	(2.7)	0.0335
From 3 to 6 minimum wages	97	(20)	3	(0.6)	
6 or more minimum wages	198	(41)	4	(0.8)	
**Race/Ethnicity**					
White	304	(64)	10	(2.1)	0.2043
Non-white	154	(32)	10	(2.1)	
**Child’s Age, years**					
0–4	147	(31)	6	(1.3)	0.4265
5–8	117	(25)	8	(1.7)	
9–12	93	(20)	4	(0.8)	
13–17	101	(21)	2	(0.4)	
**Child’s Gender**					
Male	260	(54)	15	(3.1)	0.1665
Female	198	(41)	5	(1)	

## Data Availability

Data are available upon reasonable request at the corresponding author.

## Supplementary Materials

The following are available online at https://www.mdpi.com/article/10.3390/vaccines9101115/s1, Table S1: Answers to the questionnaire about vaccinations.
