# Supplementary material for: COVID-19 Vaccine Hesitancy among Parents of Children and Adolescents Living in Brazil
Source: Vaccines (Basel). 2021 Sep 30;9(10):1115. doi: 10.3390/vaccines9101115 (PMC8540804; doi:10.3390/vaccines9101115)
Supplement: Supplementary file 1 [file vaccines-09-01115-s001.zip › vaccines-1378237-supplementary.pdf]

Table S1. Answers to the questionnaire about vaccinations.

Questions	N	(%)
Have you ever delayed having your child get a shot for reasons other than illness or allergy?		
Yes	106	(21)
No	395	(79)
Have you ever decided not to have your child get a shot for reasons other than illness or allergy?		
Yes	25	(5)
No	474	(95)
Don't Know	2	(0.4)
How sure are you that following the recommended shot schedule is a good idea for your child?		
0 = Not at all sure	0	(0)
1-5	5	(1)
6	3	(0.6)
7	3	(0.6)
8	24	(4.8)
9	27	(5.4)
10 = Completely sure	439	(88)
If you had another infant today, would you want him/her to get all the recommended shots?		
Yes	497	(99.2)
No	1	(0.2)
Don't Know	3	(0.6)
Overall, how hesitant about childhood shots would you consider your-self to be?		
1 = Not at all hesitant	317	(63)
2 = Not too hesitant	53	(11)
3 = Not sure	33	(6.6)
4 = Somewhat hesitant	30	(6)
5 = Very hesitant	68	(14)
Children get more shots than are good for them		
1 = Strongly Agree	36	(7.2)
2 = Agree	18	(3.6)
3 = Not sure	41	(8.2)
4 = Disagree	71	(14)
5 = Strongly Disagree	335	(67)
I believe that many of the illnesses shots prevent are severe		
1 = Strongly Agree	404	(81)
2 = Agree	61	(12)
3 = Not sure	10	(2)
4 = Disagree	8	(1.6)
5 = Strongly Disagree	18	(3.6)
It is better for my child to develop immunity by getting sick than to get a shot		
1 = Strongly Agree	8	(1.6)
2 = Agree	4	(0.8)
3 = Not sure	10	(2)
4 = Disagree	57	(11)

5 = Strongly Disagree	422	(84)
It is better for children to get fewer vaccines at the same time		
1 = Strongly Agree	62	(12)
2 = Agree	78	(16)
3 = Not sure	131	(26)
4 = Disagree	67	(13)
5 = Strongly Disagree	163	(33)
How concerned are you that your child might have a serious side effect from a shot?		
1 = Not at all concerned	62	(12)
2 = Not too concerned	122	(24)
3 = Not sure	49	(9.8)
4 = Somewhat concerned	138	(28)
5 = Very concerned	130	(26)
How concerned are you that any one of the childhood shots might not be safe?		
1 = Not at all concerned	166	(33)
2 = Not too concerned	109	(22)
3 = Not sure	56	(11)
4 = Somewhat concerned	81	(16)
5 = Very concerned	89	(18)
How concerned are you that a shot might not prevent the disease?		
1 = Not at all concerned	142	(28)
2 = Not too concerned	108	(22)
3 = Not sure	55	(11)
4 = Somewhat concerned	79	(16)
5 = Very concerned	117	(23)
I trust the information I receive about shots		
1 = Strongly Agree	259	(52)
2 = Agree	163	(33)
3 = Not sure	52	(10)
4 = Disagree	16	(3.2)
5 = Strongly Disagree	11	(2.2)
I am able to openly discuss my concerns about shots with my child's doctor		
1 = Strongly Agree	299	(60)
2 = Agree	127	(25)
3 = Not sure	29	(5.8)
4 = Disagree	6	(1.2)
5 = Strongly Disagree	40	(8)
All things considered, how much do you trust your child's doctor?		
0 = Do not trust at all	0	(0)
1-5	28	(5.6)
6	5	(1)
7	17	(3.4)
8	65	(13)
9	100	(20)
10 = Completely trust	286	(57)
Would you have your children vaccinated with a vaccine reported effective against COVID-19 and approved by the authorities?		
Yes	458	(91)
No	20	(4)

Don't Know	23	(4.6)
Would you have yourself vaccinated with a vaccine reported effective against COVID-19 and approved by the authorities?		
Yes	469	(94)
No	18	(3.6)
Don't Know	14	(2.8)
